# A New Method for Isolation of Interstitial Fluid from Human Solid Tumors Applied to Proteomic Analysis of Ovarian Carcinoma Tissue

**DOI:** 10.1371/journal.pone.0019217

**Published:** 2011-04-26

**Authors:** Hanne Haslene-Hox, Eystein Oveland, Kaja C. Berg, Odd Kolmannskog, Kathrine Woie, Helga B. Salvesen, Olav Tenstad, Helge Wiig

**Affiliations:** 1 Department of Biomedicine, University of Bergen, Bergen, Norway; 2 Department of Obstetrics and Gynecology, Haukeland University Hospital, Bergen, Norway; 3 Department of Clinical Medicine, University of Bergen, Bergen, Norway; Karolinska Institutet, Sweden

## Abstract

Major efforts have been invested in the identification of cancer biomarkers in plasma, but the extraordinary dynamic range in protein composition, and the dilution of disease specific proteins make discovery in plasma challenging. Focus is shifting towards using proximal fluids for biomarker discovery, but methods to verify the isolated sample's origin are missing. We therefore aimed to develop a technique to search for potential candidate proteins in the proximal proteome, i.e. in the tumor interstitial fluid, since the biomarkers are likely to be excreted or derive from the tumor microenvironment. Since tumor interstitial fluid is not readily accessible, we applied a centrifugation method developed in experimental animals and asked whether interstitial fluid from human tissue could be isolated, using ovarian carcinoma as a model. Exposure of extirpated tissue to 106 *g* enabled tumor fluid isolation. The fluid was verified as interstitial by an isolated fluid:plasma ratio not significantly different from 1.0 for both creatinine and Na^+^, two substances predominantly present in interstitial fluid. The isolated fluid had a colloid osmotic pressure 79% of that in plasma, suggesting that there was some sieving of proteins at the capillary wall. Using a proteomic approach we detected 769 proteins in the isolated interstitial fluid, sixfold higher than in patient plasma. We conclude that the isolated fluid represents undiluted interstitial fluid and thus a subproteome with high concentration of locally secreted proteins that may be detected in plasma for diagnostic, therapeutic and prognostic monitoring by targeted methods.

## Introduction

Significant efforts have been invested in the search for biological disease indicators in plasma, for diagnostic and prognostic purposes [1]. However, the high range of protein concentrations in plasma is a major obstacle in biomarker discovery using proteomics [2]. Many proteins suggested as biomarkers are relatively abundant and related to non-specific global reactions to the disease resulting in low sensitivity and specificity, thus most candidates are never translated into clinical use [3], [4]. Furthermore, proteins secreted from tumor cells and shed membrane proteins will have several orders of magnitude higher concentration in the tumor extracellular or interstitial microenvironment compared to plasma [5]. In the search for tumor specific biomarkers the focus should accordingly be on the tumor interstitial environment and the secretome and thus in the fluid phase bathing the tumor cells and the extracellular matrix elements, i.e. in the tumor interstitial fluid (TIF) [6]. To our knowledge there is, however, no technique available whereby native interstitial fluid can be isolated from solid human tumors.

Microdialysis is a technique frequently used to access the interstitial space in experimental animals and humans *in vivo*, but because of low recovery of macromolecules, fluid isolated with this technique will not reflect the protein composition of native interstitial fluid [7], [8], [9]. Attempts have been made to isolate TIF *ex vivo* and to apply this fluid as substrate for proteomic analysis [6], [10], [11], [12]. Little evidence has, however, been presented that such fluid originates solely from the interstitial fluid phase. Admixture of intracellular proteins in the isolated TIF will result in identification of proteins that will not be secreted and may thus be erroneously identified as biomarker candidates. By isolating native or undisturbed TIF without causing cellular damage, the sample will be a pre-sorted selection of proteins with characteristics that are required for proteins to be used as biomarkers (i.e. produced in the tumor), making detection of clinically relevant biomarker candidates more likely.

As pointed out in a recent review [9], access to native fluid will enable us to address a wide range of questions regarding the tumor microenvironment and tumor biology in general. As an example, we may measure interstitial fluid colloid osmotic pressure (COP), one of the determinants of transcapillary fluid exchange that also give information on sieving properties of tumor capillaries. Moreover, in native TIF we may also quantify the local production of signaling and tissue specific substances, knowledge of importance to understand how tumors develop and progress.

In the present study we asked whether a method developed for isolation of TIF in experimental animals [13] could be translated for use in human solid tumors. As an example we used ovarian carcinoma, representing the most lethal gynecological malignancy, with the majority of cases diagnosed with metastatic disease [4]. In order to verify the origin of the isolated fluid we quantified the admixture of intracellular fluid by relating the concentration of endogenous substances present predominantly in the extracellular fluid phase in isolated fluid to that of plasma, and were able to demonstrate that such admixture was negligible. We utilized the isolated fluid to determine for the first time interstitial fluid colloid osmotic pressure in a human solid malignant tumor, and could furthermore show that such fluid is a relevant substrate in a subproteome analysis when searching for tumor specific proteins. The presented new technique may serve as a useful tool in studies of the tumor cell microenvironment.

## Results

The extirpated human ovarian tumor samples (weight range 0.3–5 grams) used in the present study were from the tumor surface in an area without any apparent necrosis or inflammation, and the tumor fluid was isolated immediately after extirpation by centrifugation for 10 minutes. The tumor fluid yield after centrifugation ranged from 5 to 150 µl/g tissue, and the isolated fluid had a yellow, clear color. Fluid could be isolated from all tumor samples at 106 *g*.

### Validation by extracellular markers

**Figure 1:**
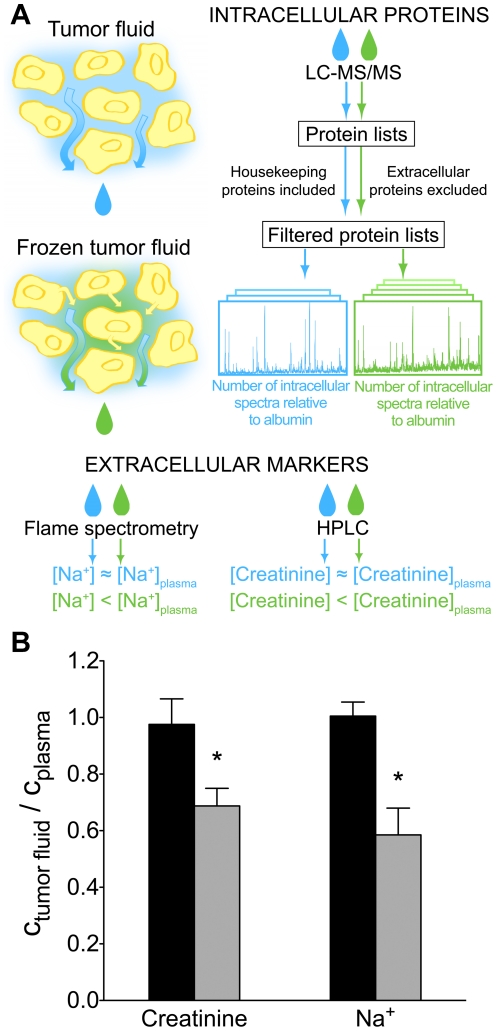
Overview of the tumor fluid validation assay. A: Illustration of the principle for validation of tumor fluid as interstitial fluid. The concentrations of the extracellular markers, Na^+^ and creatinine were compared in isolated tumor fluid and plasma. When the isolated fluid derives solely from the interstitial space, the concentrations of the markers are equal to that in plasma, while addition of intracellular fluid will result in a lower tumor fluid:plasma ratio. To assess the presence of intracellular proteins, mass spectrometric analysis was used, the resulting protein lists were filtered based on known house keeping and extracellular proteins and the change in the number of spectra for intracellular proteins relative to albumin was used as an indicator of intracellular protein contamination. B: Measured tumor fluid:plasma ratio for creatinine and Na^+^ for tumor fluid (black) and frozen tumor fluid (grey). Values are mean ± SEM. *: Significantly different from tumor fluid (p<0.05) (Mann-Whitney test).

In order to address whether the tumor fluid derived from the extracellular space only or had admixture of intracellular fluid, we designed an assay (Figure 1A) based on measuring the concentration of two endogenous substances, creatinine and Na^+^. These substances are present predominantly in the extracellular fluid phase and should therefore have similar concentrations in plasma and interstitial fluid [14], [15], [16].

The tumor fluid:plasma ratios were 0.98±0.09 for creatinine (n = 7) and 1.00±0.04 for Na^+^ (n = 7) (Figure 1B). Dilution with intracellular fluid would result in a ratio lower than unity, and neither the creatinine nor the Na^+^ values were significantly different from 1.0 (One-sample *t*-test).

As an additional measure to study how cellular rupture and release of intracellular proteins affected the creatinine and Na^+^ measurements we exposed frozen samples to 955 *g*, a procedure known to cause such release [13], and these samples are referred to as frozen tumor fluid. These samples gave a frozen tumor fluid:plasma ratio that was significantly lower than 1.0 for both substances (Mann-Whitney, p<0.05) averaging 0.69±0.06 (n = 4) for creatinine and 0.59±0.09 (n = 6) for Na^+^ (Figure 1B), suggesting that intracellular fluid with low concentrations of Na^+^ and creatinine had been added to the centrifugate.

**Figure 2:**
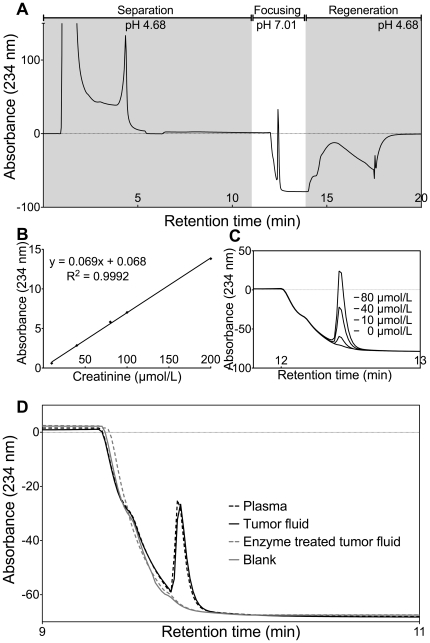
Measurement of creatinine by HPLC. A: The complete chromatogram from HPLC creatinine analysis. Deproteinized samples were injected on two strong cation exchange columns in series at pH 4.68, where creatinine will be positively charged and retained. TCA and other contaminants are not retained at this pH and were washed out in the first five minutes, while creatinine was passed into a third column for further separation and fixation. By changing the buffer pH to 7.01 in the third column the creatinine molecule was rendered neutral, reducing retention and thus producing a sharp and well-defined peak with absorbance at UV 234 nm. After a focusing step the buffer pH changes back and the columns were regenerated. B: Calibration curve for creatinine with standard concentrations 0, 10, 40, 80, 100 and 200 mM. C: Chromatograms for creatinine standards 0, 10, 40 and 80 µmol/L used for calibration. D: By analyzing tumor fluid and plasma samples before and after enzyme hydrolysis by creatininase and creatinase the method's specificity could be assessed. The chromatograms from analysis of plasma, tumor fluid before and after enzyme hydrolysis, and a blank run are shown.

The HPLC approach for determining creatinine concentration and separation of creatinine from interfering chromogens is shown in Figure 2A–C. To address possible interference in the creatinine assay by chromogens [15] the enzymes creatininase and creatinase were used to remove creatinine from the sample before analysis. The signal after enzyme hydrolysis was minimal (Figure 2D) suggesting that chromogens did not affect the creatinine measurement.

### Validation based on intracellular proteins

In order to determine the admixture of intracellular proteins that may result from the handling of the tissue samples and the centrifugation procedure, freshly isolated and frozen tumor fluid were compared by a mass spectrometry (MS) approach without prior fractionation. To study proteins that are mainly intracellular, i.e. housekeeping proteins, the protein lists were filtered. First, proteins identified in plasma and proteins assigned to the gene ontology (GO) category cellular component “Extracellular” were excluded. Second, proteins present in a list of housekeeping genes predicted by a Naive Bayes classifier were included [17]. Fifteen proteins matched the filtering criteria, and they are given in Table S1. The sum of spectra for these housekeeping proteins was normalized by the number of albumin spectra identified in tumor fluid and frozen tumor fluid, respectively. We found that the number of spectra representing intracellular proteins increased by 80% in frozen tumor fluid compared to the tumor fluid, suggesting a significant addition of intracellular proteins to the frozen tumor fluid.

### COP

**Figure 3:**
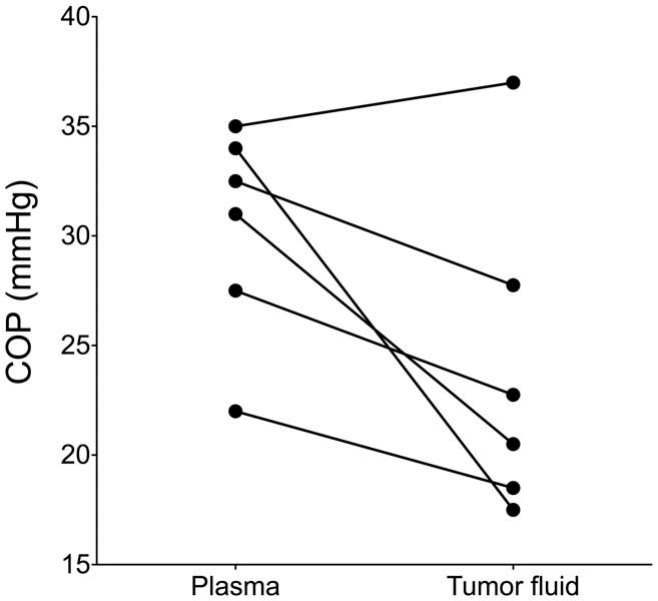
Colloid osmotic pressure (COP) in plasma and tumor fluid samples. COP was measured in paired plasma and tumor fluid samples using a colloid osmometer with a 30 kDa cut off-membrane. Corresponding pressure measurements in plasma and tumor fluid have been connected.

Since the isolated tumor fluid obtained from the tumor was undiluted, we were able to measure the COP in interstitial fluid of human ovarian tumors (Figure 3). The COP averaged 24.0±3.0 mmHg in tumor fluid (n = 6), significantly different from the corresponding pressure in plasma of 30.3±2.0 mmHg (n = 6) (p<0.05, Wilcoxon matched-pairs signed rank test), thus resulting in a tumor fluid:plasma COP ratio of 0.79±0.08.

### Characterization of proteins in tumor fluid compared to plasma and ascites

**Figure 4:**
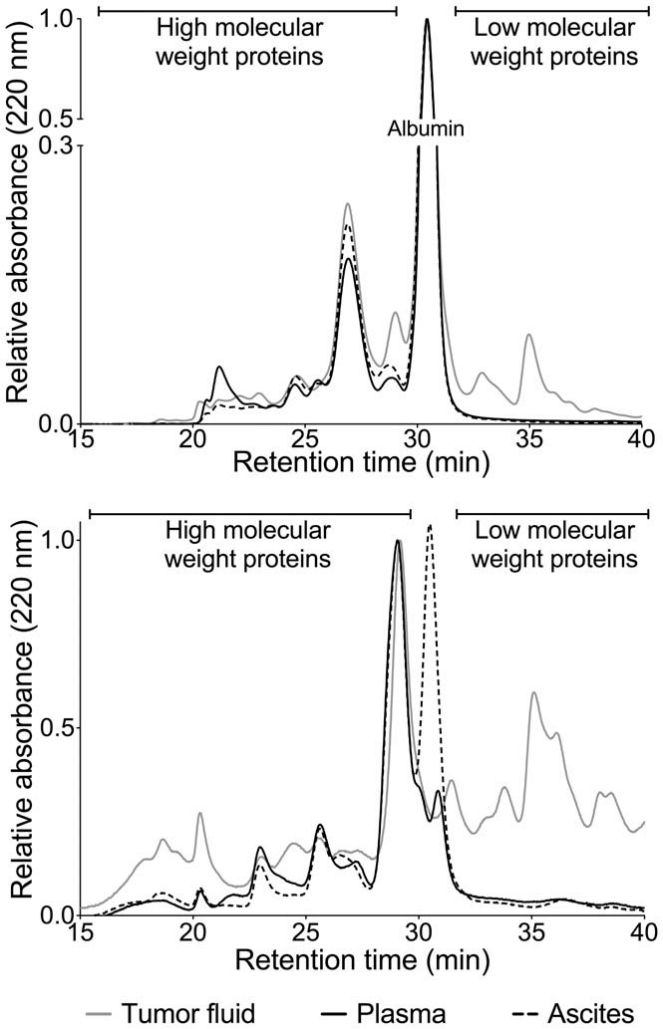
Chromatographic evaluation of tumor fluid, plasma and ascites by size-exclusion chromatography. A: Size exclusion-chromatography of native tumor fluid, plasma and ascites, results are normalized in respect to albumin. As expected, albumin was a major constituent, and all three samples were dominated by major plasma proteins. The chromatogram for tumor fluid indicates that there were some low molecular weight proteins present in tumor fluid that were not found in ascites or plasma. B: After immunoaffinity depletion of the 14 most abundant plasma proteins, the ascites and plasma samples were still similar, except from an albumin peak for ascites that can indicate incomplete depletion of albumin. Tumor fluid reveals large differences in the protein composition compared to plasma and ascites, indicating the presence of tumor specific proteins in tumor fluid. Results were normalized in respect to the peak at a retention time of 29 minutes.

To characterize the tumor fluid with respect to protein composition, tumor fluid, plasma and ascites were analyzed by size-exclusion chromatography before and after immunodepletion of the 14 most abundant plasma proteins. Before immunodepletion all samples were dominated by the presence of high abundance plasma proteins with albumin as the most marked peak with retention time of 30.5 minutes (Figure 4A), but tumor fluid also presented with a higher signal compared with ascites and plasma showing that there were more proteins eluting in the low molecular weight area after albumin. The chromatograms were normalized to albumin. After immunodepletion the overall protein composition in the samples changed (Figure 4B). At a retention time of 29 minutes, all three samples had a dominating peak, that we have shown is concurrent with hemopexin (unpublished observations), and all three chromatograms were normalized to this peak. The ascites sample still had a marked peak at the retention time for albumin at 30.5 minutes, but was otherwise similar to plasma. In contrast, tumor fluid differed significantly from plasma and ascites both in the first part of the chromatogram, at 15 to 20 minutes, and after 30 minutes. This analysis showed that the TIF contained significant amounts of potential tumor specific proteins that were not detectable in plasma or ascites and may thus serve as an improved substrate for proteomic analysis.

### Proteomic analysis

**Figure 5:**
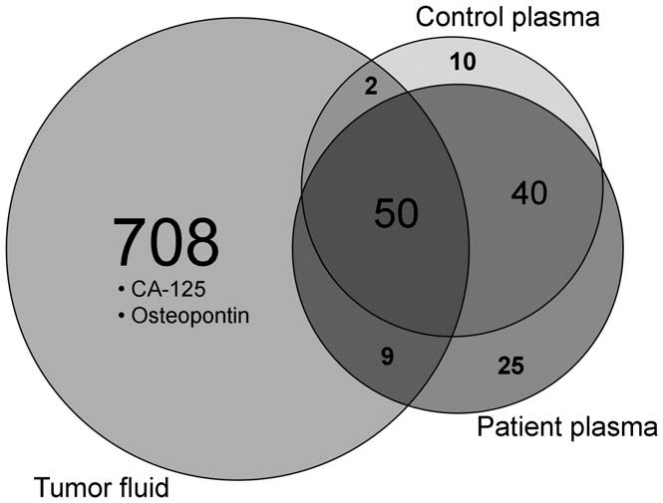
Overview of proteomic results for tumor fluid compared to plasma. Pooled plasma and tumor fluid samples of three patients with ovarian cancer and a control pool taken from five women operated for suspected ovarian carcinomas later shown to be benign were immunoaffinity depleted, fractionated by reversed phase and strong cation exchange chromatography at protein level and nano-reversed phase liquid chromatography at peptide level before analysis by tandem mass spectrometry. Venn-diagram of the three proteomes identified from tumor fluid (left), patient plasma (lower right) and control plasma (upper right). Analyzing patient plasma compared to control plasma yielded few new proteins, while the tumor fluid contains a large amount of proteins not detected in plasma.

The tumor fluid was investigated as a starting point for biomarker discovery using a proteomic approach. The proteomic analysis resulted in identification of 769 proteins in the tumor fluid, 124 in patient plasma and 102 in control plasma (Figure 5). Protein lists are given in Table S2. The two plasma proteomes had 66% of the proteins identified in common, an overlap that is expected with two technical replicates of the same sample [18], [19], whereas more than six times as many proteins were identified in tumor fluid compared to plasma, underlining the difficulty to find disease-specific changes in the plasma proteome. Among the proteins found in both patient plasma and tumor fluid we identified the established biomarker CA-125 (Q8WXI7), and among the 708 proteins detected only in tumor fluid the new biomarker candidate Osteopontin (P10451) [20], [21] was also found, as well as a large number of proteins that have yet to be explored as biomarker candidates.

The protein composition according to GO cellular component may indicate the origin of the proteins found in tumor fluid and patient plasma. For patient plasma 90.3% and 51.6% of the proteins identified were annotated with the GO category “Extracellular” and “Cytoplasm” respectively, whereas tumor fluid had 27.8% of the proteins annotated as “Extracellular” and 83.4% as “Cytoplasm”.

**Figure 6:**
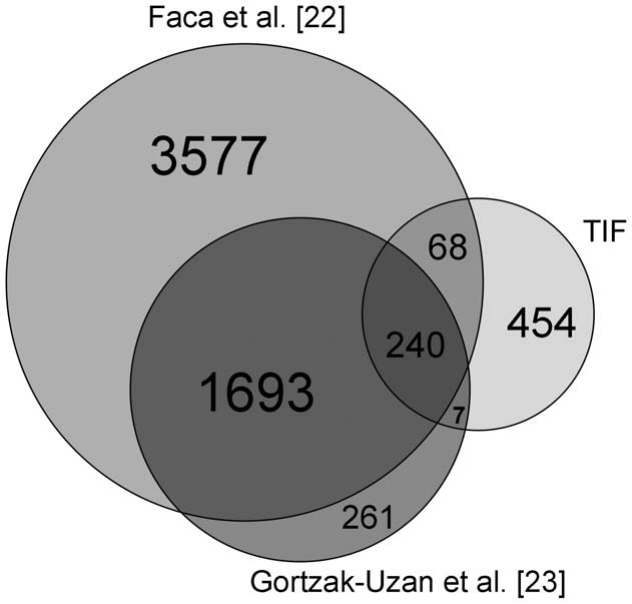
Comparison of tumor fluid proteome with published protein data. The proteins found in tumor fluid in the present study (right) compared to proteomes presented for ascites by Gortzak-Uzan et al. [23] (lower left) and ovarian cancer cell line cultures of Faca et al. [22] (upper left). In ascites there were 268 proteins in addition to the proteins found by proteomic analysis of ovarian cancer cell lines cultures, while in the tumor fluid proteome, although with a much lower total number of proteins identified, there were 454 proteins that were neither detected in ascites nor in cell line cultures.

In an attempt to investigate whether our method of interstitial fluid isolation has the potential to reveal new biomarker candidates that is not identified with alternative methods, we compared the 769 proteins found in TIF with data from previous in-depth proteomic analysis of ovarian cancer cell line cultures [22] and ascites deriving from ovarian cancer patients [23]. Interestingly, we were able to detect 454 proteins in TIF that were not found in the other proteomes (Figure 6), suggesting that access to the tumor subproteome through TIF represents an important new source for biomarker candidate proteins.

## Discussion

Here we have presented a method to isolate native interstitial fluid from a solid human tumor and verified that the isolated fluid derives from the extracellular fluid phase. Even though the interstitial fluid is an important element of the tissue microenvironment, TIF has been coined as a “misconsidered component of the internal milieu of a solid tumor” [24]. The reason is most likely a paucity of methods for TIF isolation [9], and we have thus provided a new tool to remedy this situation.

The critical question in our work is whether fluid isolated by centrifugation is representative for the interstitial fluid. Previously we have addressed this issue in animal studies by using the extracellular tracer ^51^Cr-EDTA [13], whereas an alternative approach is necessary for studies of human tissue. We therefore used two endogenous substances, creatinine and Na^+^ to assess if the tumor fluid is representative for TIF.

Creatinine originates from degradation of creatine, which mainly occurs in skeletal muscle tissue harboring 98% of the bodily creatine [15], diffuses out from the cells and into plasma, and is present in equilibrium in the extracellular fluids of the body [15].

Na^+^ is in equilibrium between plasma and the interstitial fluid [14], [16], and has a relatively high concentration in the extracellular (145 mM) as compared with the intracellular fluid phase (4 mM). Both substances are, however, restricted from entering the cell. Our finding that the ratios for the concentrations of Na^+^ and creatinine in tumor fluid compared to plasma were not significantly different from 1.0, suggests that the isolated fluid is not diluted by intracellular fluid.

Obviously, the isolated fluid derives from the entire extracellular fluid phase, and will therefore include some plasma. Using ^125^I-labeled serum albumin, we have assessed this fraction in fluid isolated by centrifugation from chemically induced mammary carcinomas in rats [13], another solid epithelial tumor. At a G-force similar to what was applied in the present study, we found that ∼5% of the isolated fluid derived from plasma. Similar studies are for obvious reasons not feasible in humans, but if we assume that these tumors have comparable physicochemical properties we may conclude that the fraction deriving from plasma is negligible and thus that the isolated fluid represents TIF.

Having concluded that the tumor fluid is representative for TIF, the finding of intracellular proteins in the same fluid shows that there are some intracellular proteins in TIF. Furthermore, our GO analysis indicates a larger fraction of extracellular proteins in plasma than in tumor fluid. Still, as the tumor fluid originates in the tissue, and plasma is a global extracellular fluid, a higher fraction of cytoplasmic proteins in tumor fluid than in plasma is to be expected. These proteins may derive from cellular catabolism *in vivo*, which will affect the detection of intracellular proteins, but not affect the creatinine- and Na^+^-ratios. This is consistent with the finding of a large number of peptides derived from intracellular sources in prenodal lymph [25], [26], which is representative for tissue interstitial fluid [9]. Intracellular proteins will also leak to plasma, as evidenced by a significant amount of such proteins in human serum [27]. It is also likely that the TIF contains peptides deriving from extracellular matrix elements cleaved by matrix metalloproteases known to be active in the tumor interstitium [28], [29]. Proteins deriving from necrotic areas may potentially contaminate the sampled interstitial fluid. Although we cannot exclude such contamination completely, there are several reasons why we think this effect is small. When we isolated tumor tissue for centrifugation, necrotic areas were avoided. Furthermore, fluid isolated from necrotic areas of the tumor will likely lead to leakage of intracellular fluid with low Na^+^ and creatinine to the microenvironment, and thus result in reduced tumor fluid:plasma ratios for these two extracellular markers. Assessing the distribution of proteins in cellular components as defined by GO may indicate that there is a large amount of intracellular proteins present. However, preliminary results from proteomic analysis of interstitial fluid isolated from healthy ovarian tissue (unpublished) gave a GO distribution highly similar to that found in TIF, again supporting our assumption that the observed protein pattern is not a result of tumor necrosis.

Intracellular contamination has to be considered when interpreting proteomic data originating from frozen samples, e.g. from biobanks, which may be used for targeted proteomics. If, however, frozen tissue is used in a screening phase, this will result in the detection of intracellular non-secretome candidate proteins.

Other methods like nipple aspirates and ductal lavage fluid have been proposed for sampling of tumor subproteomes (for review see [30]), and in-depth proteomic analyses have been performed on peritoneal fluid [31] and ascites [23], [32] for ovarian cancer. Ascites fluid may accumulate in the peritoneal cavity of ovarian cancer patients in advanced stages [3]. Such accumulation is a result of malignant cells secreting proteins that cause neovascularization, increased fluid filtration and lymphatic obstruction leading to build-up of serum-like fluid within the abdomen [32]. That this fluid is similar to plasma was also shown by our experiments, but we could furthermore demonstrate that ascites deviates strongly from TIF with respect to protein composition as illustrated by size-exclusion chromatography. Thus, tumor specific proteins are likely up-concentrated in tumor fluid compared to both plasma and ascites. Moreover, since ascites accumulates at an advanced stage, such fluid may, as suggested by Gortzak-Uzan and coworkers [23], not be so useful to detect early disease protein expression signatures but rather to predict outcome and treatment response. TIF sampling as presented here will, however, give a protein signature that in addition to being representative for an earlier stage of the disease also enable us to quantify microenvironmental proteins.

Another method used for sampling of interstitial fluid from human tumors is tissue elution [10]. By this method, fresh biopsies obtained from women with invasive breast cancer are cut into small pieces (1–3 mm^3^) and eluted in PBS. The supernatant is collected after 1 hour and named TIF [10]. Sectioning of cell-rich tumors into small pieces may result in admixture of intracellular fluid. The major problem with this tissue elution method is thus to determine if the identified peptides and proteins are representative for the interstitial fluid or have entered the eluate from the intracellular compartment during the PBS-equilibration period. Sun et al. [11] addressed intracellular protein contamination in eluted TIF by Western blot. Based on the fact that they were unable to find four organelle specific proteins, they concluded that intracellular proteins did not contaminate the TIF. Evidently, other intracellular proteins may enter the extracellular phase during equilibration, since there is a significant fraction of intracellular proteins even in normal serum [27], suggesting that the finding of such proteins *per se* cannot be used as evidence of intracellular fluid contamination.

Access to native, undiluted TIF has additional advantages. Eluate will not as centrifugate reflect absolute concentration in TIF of proteins and signaling substances, of importance for e.g. protein expression and transcapillary fluid balance studies. The determination of COP will only be feasible in undiluted samples, and to our knowledge we are the first to report COP in human tumor tissue, underlining the advantage of tumor fluid isolated by centrifugation. The measured tumor fluid:plasma COP ratio of 0.79 is higher than in human skin [33], but consistent with earlier findings for mammary tumors in rats [13]. A ratio <1.0 indicates that there is some sieving of proteins at the capillary wall of tumors. Furthermore, this observation suggests that uptake of therapeutic agents in tumors can be improved by using solutions with high COP to lower interstitial fluid pressure as suggested by experiments in mice [13], [34].

As part of the validation of the method we performed a proteomic analysis of the tumor fluid and found a sixfold increase in the number of detected proteins compared to plasma, pointing to TIF as a rich source for tumor specific proteins with biomarker potential. Moreover, a comparison with published in-depth proteomes for ovarian cancer derived from cell cultures and ascites [22], [23] showed that analysis of TIF and thus the native tumor microenvironment resulted in the identification of a significant number of additional proteins not found in these earlier studies, emphasizing the value of our sampling approach.

One may argue that we present proteomics data on pooled fluid from few patients and thus question the general validity of the results. Our aim was to present an approach to isolate native TIF, but our data may also be considered as an initial phase of discovery proteomics. In this phase the main goal is to accumulate a library of proteins that may serve as candidates for validation strategies later [35]. Since the fractionation process is extensive and requires large sample volumes, individual tumor fluid samples were pooled to serve as a source for such an ovarian cancer library based on tumor fluid and plasma. The method of pooling samples has been presented as an approach to reduce the effects of biological variation and with the additional benefit of a great reduction in the total number of samples that has to be analyzed [36], [37]. Moreover, pooling of samples reduces the influence of individual changes in protein composition and favors those proteins present in all the individual samples that may serve as more robust biomarker candidates. Further mining and validation studies will clearly be needed to establish the potential roles for the identified proteins as novel biomarker candidates.

To summarize, we have presented a centrifugation technique that can be used to isolate undiluted fluid that qualitatively and quantitatively reflects human ovarian carcinoma interstitial fluid. The method can be adapted for application in other solid tumors and be used to address a wide range of questions regarding the tumor microenvironment and tumor biology. Additionally, as suggested here, it can be used for identification of candidate proteins for diagnostic and prognostic use as well as targets for molecular imaging and therapy.

## Materials and Methods

The research protocol has been approved by the Norwegian Data Inspectorate (Protocol # 961478-2), Norwegian Social Sciences Data Services (Protocol # 15501) and the local ethical committee (Protocol ID REKIII nr. 052.01). All samples were collected after obtaining the patients' written informed consent. The work conformed to the standards set by the latest revision of the Declaration of Helsinki.

### Sample collection

Samples from extirpated human ovarian tumors were collected from the Department of Obstetrics and Gynecology at Haukeland University Hospital. The samples were taken from the tumor surface in an area without any apparent necrosis or inflammation, and ascites fluid was collected in patients presenting with fluid in the abdominal cavity. Blood samples were collected from the patients 1–2 days before surgery and EDTA was used as the coagulation agent. Tumor fluid was isolated immediately after extirpation by centrifugation as described earlier [13] at 106 *g* for 10 minutes, and fluid without erythrocytes were collected and stored −80°C until further processing. Fluid that was isolated from fresh tissue is referred to as tumor fluid. As a positive control for admixture of intracellular fluid, a piece of the tumor sample was frozen without centrifugation, and subsequently thawed and centrifuged at 955 *g* for 10 minutes, referred to as frozen tumor fluid. Ascites fluid was centrifuged to remove the cellular fraction, and cell free ascites fluid was extracted. The fluid samples were stored at −80°C until further processing. The sample validation strategy is outlined in Figure 1A.

### Creatinine analysis

The concentration of creatinine was measured in individual samples (n = 7) by HPLC on an Agilent HPLC system, using the principle presented by Ambrose et al. [38]. Ten µl samples were deproteinated by 90 µl 5% TCA and spun at 20 000 *g* for 10 minutes to remove precipitated proteins, 98 µl of supernatant was pipetted off, and 50 µl supernatant was injected in the HPLC system with 20 mM sodium acetate buffer (pH 4.68). At this pH creatinine, with a pKa of 5.02, is positively charged and has some retention on two strong cation exchange (SCX) columns. A Bio-Monolith SO_3_ column (5.2×4.95 mm, Part no.: 5069-3637, Agilent Technologies, Santa Clara, CA) in series with a Resource S column (1 ml, Code no. 17-1178-01, GE Healthcare, United Kingdom) were used for separation of creatinine from TCA and other contaminants. Further separation was done with a Proswift SCX-1S Analytical column (4,6×50 mm, 066765, Dionex, Sunnyvale, CA) and by changing the buffer to 10 mM phosphate buffer (pH 7.01) in the third column the creatinine molecule is rendered neutral, reducing the retention in the column and focusing it, thus producing a sharp and well-defined peak with absorbance at UV 234 nm.

The specificity of the method was tested by analyzing plasma and tumor fluid before and after enzyme hydrolysis by creatinase (EC 3.5.3.3, 500 U, Sigma-Aldrich, St. Louis, MO) and creatininase (EC 3.5.2.10, 1000 U, Sigma-Aldrich) as suggested by Paroni et al. [39]. The lyophilized enzymes were reconstituted in 100 mM phosphate buffer (50 mM Na_2_HPO_4_ + 50 mM NaH_2_PO_4_) to a final concentration of 50 and 90 U/ml for creatininase and creatinase, respectively. Ten µl of sample was incubated at 25°C overnight with 5 µl of each enzyme added. The following day the sample was deproteinated by adding 80 µl of 5% TCA and processed further as described above.

### Sodium analysis

The concentration of Na^+^ was measured in individual samples (n = 7) by an AAnalyst 200 flame spectrometer (PerkinElmer, Waltham, MA). The instrument was calibrated using solutions with Na^+^-concentrations of 10, 20, 50, 100 and 200 mM that were further diluted 1∶1000 in 0.65% HNO_3_. Five µl of sample was diluted in 10 ml 0.65% HNO_3_ and the concentration was measured in triplicate. All sample measurements were in the linear area of the calibration curve, between 10 and 100 mM.

### COP

COP was determined in tumor fluid and plasma for individual samples (n = 6) in a colloid osmometer using a membrane with cutoff 30 kDa and equipped with a transducer as described in detail in an earlier publication [40].

### Sample fractionation and proteomic analysis

For proteomic analysis equal volumes of tumor fluid from three tumor samples and the respective plasma samples were pooled and an overview of the patient samples used is given in Table 1. None of the patients received neoadjuvant therapy, and all had a preoperational CA-125 concentration of more than 10 times higher than the cut-off concentration of 35 U/mL. Pooled plasma from five women operated for suspected cancerous growths later shown to be benign served as control. The pooled samples were depleted of the 14 most abundant plasma proteins using a Hu-14 Multiple Affinity Removal Column (4,6×50 mm, 5188-6557, Agilent Technologies), in accordance with the manufacturer's recommendations.

**Table 1:** Overview of subgroup and stage of the patient samples used for proteomic analysis.

Patient no.	Age at diagnosis	Histology	Stage	CA-125 (U/ml)
1	47	Serous borderline ovarian tumor	IIC	710.3
2	72	Serous adenocarcinoma	IIIC	559.3
3	79	Serous adenocarcinoma	IIIC	361.4

The flow-through fraction from the immunodepletion was denatured by adding 0.48 g/ml urea and 13 µl/ml neat acetic acid and the samples were injected onto a macroporous Reversed-Phase (mRP) C18 Column (4.6×50 mm, 5188-5231, Agilent Technologies) and fractionated, following recommendations by the manufacturer.

Fraction collection was started at retention time 4 minutes. Twenty-three mRP-fractions of 1.8 ml were collected, evaporated (Eppendorf Concentrator 5301) and subsequently diluted in 100 mM ammonium bicarbonate, denatured and digested by trypsin in accordance with a protocol provided by Agilent Technologies (www.chem.agilent.com; publication no. USHUPO3), desalted by C-18 spin columns (Pepclean C-18, 89870, Pierce, Rockford, IL) and analyzed by liquid chromatography coupled with tandem MS (LC-MS/MS) with an Agilent 1100 LC/MSD Trap XCT Plus system with a HPLC-Chip/MS interface. The HPLC-Chip contained a 0.075×43 mm ZORBAX 300SB C18 5- µm column and an integrated 9 mm 160 nL enrichment column packed with the same material (part no. G4240-63001 SPQ110, Agilent Technologies). In addition, tumor fluid and frozen tumor fluid from two patients were digested by trypsin and analyzed by LC-MS/MS in triplicate without any prior separation. The enzyme-to-protein ratio used in the digestion protocol was 1∶35. For two-dimensional LC-MS/MS, each mRP fraction was separated further offline on a SCX column (ZORBAX BioSCX Series II, 0.8 mm×50 mm, 3.5 µm; strong cation exchanger, 5065-9942, Agilent Technologies) before MS analysis. Spectrum Mill MS proteomics workbench software (Rev A.03.02.060, Agilent Technologies) was used to process the MS spectra and identifications were accepted with at least two unique peptides and with a score of more than 13. Proteincenter (Software Version 3.0.4, Proxeon Bioinformatics A/S, Odense, Denmark) was used for processing the MS results, and Graphpad Prism was used for statistical analysis (Software version 5.0, Graphpad Software Inc, La Jolla, CA). Results are presented as mean ± SEM.

### Size-exclusion chromatography

Pooled samples of control plasma (n = 5), ascites (n = 9) and tumor fluid (n = 6) were analyzed by size exclusion chromatography before and after immunoaffinity depletion on a TSKgel SW2000 (4.6×300 mm, 18674, Tosoh Bioscience, Tokyo, Japan) and a TSKgel SW3000 (4.6×300 mm, 18675, Tosoh Bioscience) coupled in series. Samples that were not immunodepleted were diluted 1∶100 in mobile phase buffer (0.1 M Na2SO4 in 0.1 M phosphate buffer, pH 6.8). Immundepleted samples were not diluted, and 20 µL of all samples were injected on the HPLC system for separation.

## Supporting Information

Table S1 **Intracellular proteins.** Number of spectra identified for albumin and 15 selected intracellular proteins.
(PDF)

Table S2 **Information on identified proteins.** Summary of identified proteins, with detailed information, in tumor fluid, patient plasma and control plasma with a Spectrum Mill score higher than 13 and at least two peptides.
(XLS)
