# Supplementary material for: A New Method for Isolation of Interstitial Fluid from Human Solid Tumors Applied to Proteomic Analysis of Ovarian Carcinoma Tissue
Source: PLoS One. 2011 Apr 26;6(4):e19217. doi: 10.1371/journal.pone.0019217 (PMC3082557; doi:10.1371/journal.pone.0019217)
Supplement: Table S1 — Intracellular proteins. Number of spectra identified for albumin and 15 selected intracellular proteins. (PDF) [file pone.0019217.s001.pdf]

Table S 1: Number of spectra identified for albumin and 15 selected intracellular proteins

Protein name	NCBI accession number	Frozen tumor fluid	Tumor fluid
Albumin	28590	141	247
Actin, cytoplasmic 2	4501887	14	23
heat shock 70kDa protein 1A	62089222	6	1
Glyceraldehyde-3-phosphate dehydrogenase	31645	15	21
Protein kinase C inhibitor protein 1	30354619	5	6
Ubiquitin-activating enzyme E1	23510338	6	6
Glutathione S-transferase P	2204207	6	5
Cofilin 1	5031635	0	3
Elongation factor 1-alpha	158258715	9	1
Junction plakoglobin	194373749	0	0
S100 calcium binding protein A11	5032057	13	2
histone 1, H4e	119575948	0	1
Histone H2A type 1B	32111	0	2
Heterogeneous nuclear ribonucleoprotein D	119626277	2	0
Histone H2B type 1-M	45768638	0	2
Immunoglobulin heavy chain variable region	16076347	0	1
Sum of intracellular spectra		76	74
Intracellular spectra normalized on albumin		0,54	0,30
